# Supplementary material for: Event boundaries shape temporal organization of memory by resetting temporal context
Source: Nat Commun. 2022 Feb 2;13:622. doi: 10.1038/s41467-022-28216-9 (PMC8810807; doi:10.1038/s41467-022-28216-9)
Supplement: Supplementary file 1 — Supplementary Information [file 41467_2022_28216_MOESM1_ESM.pdf]

Supplementary Information

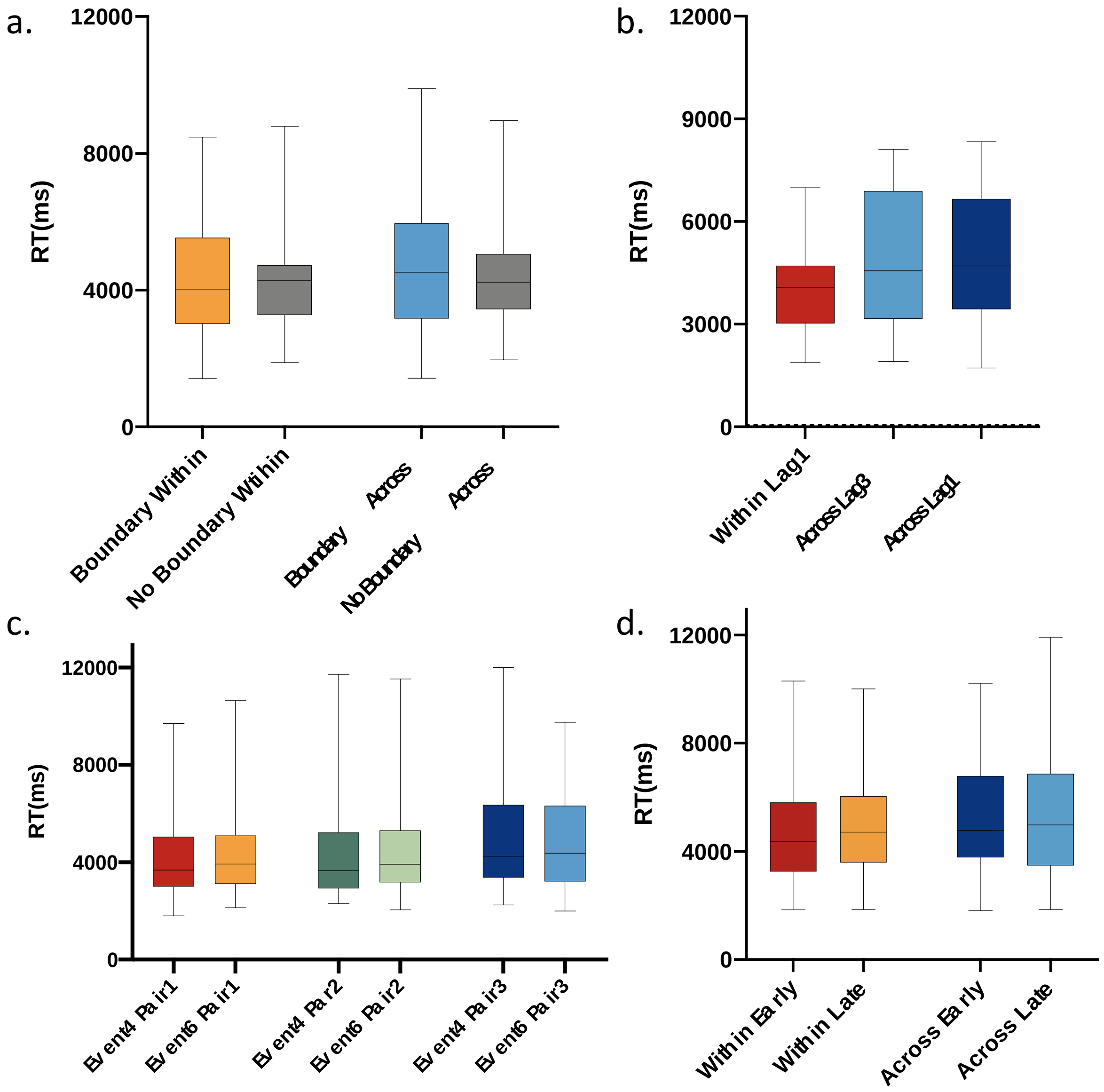

Supplementary Figure 1: **Box plots of reaction time (RT) on recency judgments for Experiments 1-4 (a-d).** For Experiment 1 (a), a two (Condition: boundary vs. no boundary) by two (Pair Type: [matched] within vs. [matched] across-event pairs) within-measures ANOVA (n=26) revealed a significant interaction between Condition and Pair Type ($F(1,25)=10.44$, $p=0.0034$, $\eta^2=0.4588\%$, 95% CI = -750.2 to -166.2). Simple effect analyses revealed no significant difference between within and across-event pairs in the no boundary condition ($t(25)= 0.08058$, $p= 0.9364$, $q>0.05$, FDR corrected for multiple comparisons), but a significant difference between within and across-event pairs in the boundary condition ($t(25)=4.651$, $p<0.001$, two-sided, $q< 0.05$, FDR corrected for multiple comparisons). For Experiment 2 (b), a one way (Pair Type: within Lag1 vs. across Lag3 vs. across Lag1) within-measures ANOVA

(n=27) revealed a significant main effect of Pair Type ($F(1.467, 38.13) = 14.85$, $p < 0.001$, $R^2=0.3636$, 95% CI = -750.2 to -166.2). Simple effect analyses showed that RT was significantly faster for within Lag1 than both across Lag3 ($t(26)= 4.158$, $p<0.001$, two-sided, $q< 0.05$, FDR corrected for multiple comparisons) and across Lag1 ($t(26)=4.115$, $p<0.001$, two-sided, $q< 0.05$, FDR corrected for multiple comparisons), but no significant difference was found between across Lag3 and across Lag1 ($t(26)= 0.4044$, $p=0.6892$, two-sided, $q> 0.05$, FDR corrected for multiple comparisons). For Experiment 3 (c), a two (Condition: Event 4 vs. Event 6) by three (Pair Type: pair type 1 vs. 2 vs. 3) within-measures ANOVA (n=32) revealed a significant main effect of Pair Type ($F(1.527,47.33)=10.53$, $p<0.001$, $\eta^2= 0.1460\%$). No other significant effect was found. For Experiment 4 (d), a two (Pair Type: within vs. across) by two (Position Type: early vs. late) within-measures ANOVA (n=30) revealed a significant main effect of Pair Type ($F(1,29)=13.6$, $p<0.001$, $\eta^2= 1.343\%$); no significant main effect of Position Type was found ($F(1,29)=0.5447$, $p=0.4664$, $\eta^2= 0.04690\%$). The boxes show the inter-quartile range (IQR) and the median. Whiskers in box plots represent the minimum and maximum in the dataset. Source data are provided as a Source Data file.

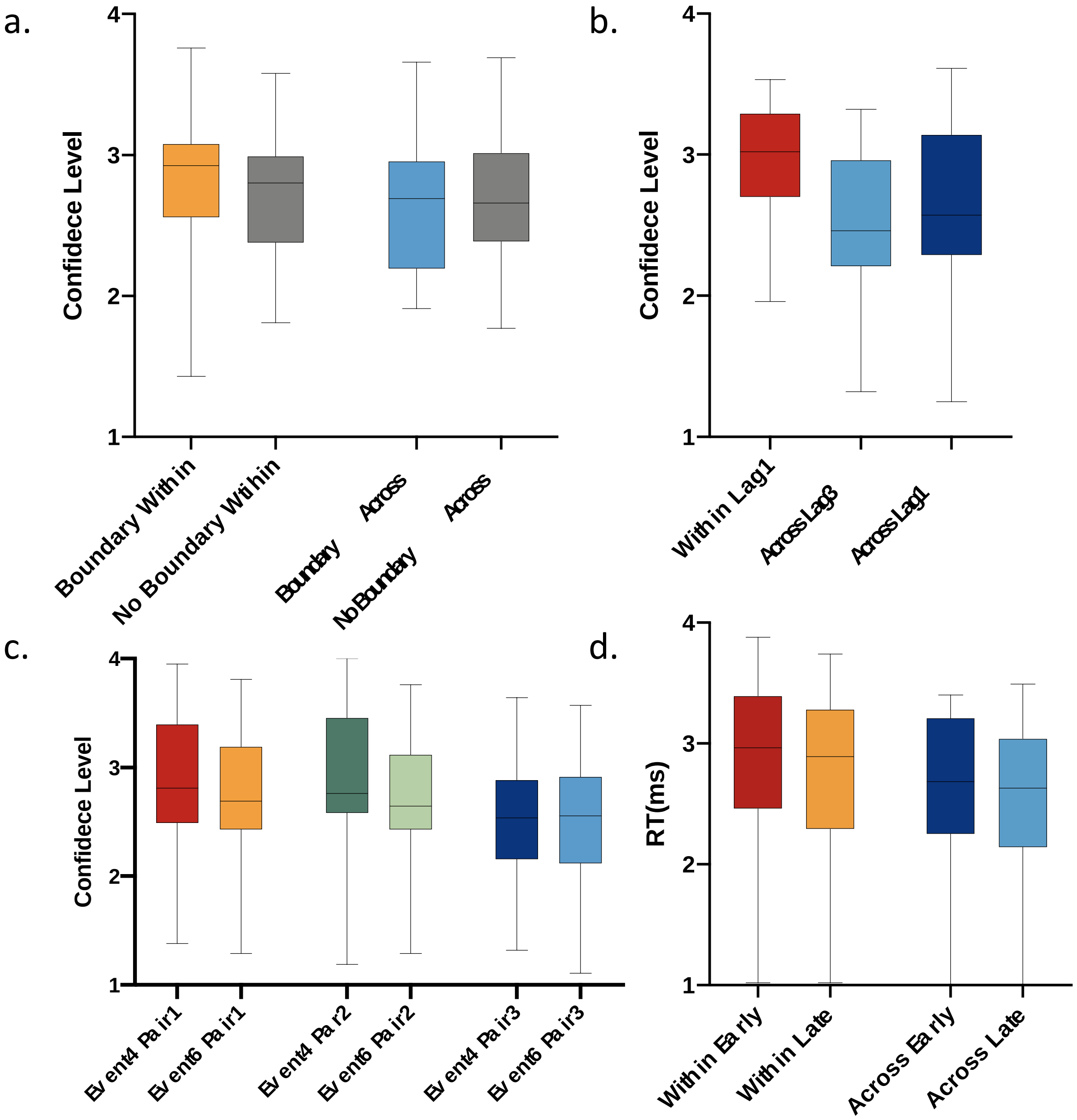

Supplementary Figure 2: Box plots of the score of confidence rating on recency judgments for Experiments 1-4 (a-d). For Experiment 1 (a), a two (Conditions: boundary vs. no boundary) by two (Pair Type: [matched] within vs. [matched] across-event pairs) within-measures ANOVA (n=26) revealed a significant main effect of Pair Type (F(1,25) = 8.989, p=0.0061, η^2=0.4588%, 95% CI = 0.04100 to 0.2209). No significant main effect of Condition was found (F (1, 25) = 0.2842, p=0.5987, η^2=0.0636%, 95% CI = -0.07103 to 0.1206) nor a significant interaction between Condition and Pair Type (F(1, 25) = 2.624, p=0.1178, η^2=0.4238%, 95% CI = -0.2909 to 0.03477). For Experiment 2 (b), a one way (pair type: within Lag1 vs. across Lag3 vs. across Lag1) (n=27) revealed a significant main effect (F(1.645, 42.78) = 14.68, p < 0.001, R^2=0.4175). Simple effect analyses showed that confidence was significantly higher for within Lag1 than both across Lag3 (t(26)= 4.602, p<0.001, two-sided, q<0.05, FDR corrected for multiple

comparisons) and across Lag1 ($t(26)= 3.664$, $p=0.0011$, two-sided, $q<0.05$, FDR corrected for multiple comparisons), but no significant difference was found between across Lag3 and across Lag1 ($t(26)= 1.524$, $p=0.1395$, two-sided, $q>0.05$, FDR corrected for multiple comparisons). For Experiment 3 (c), a two (Condition: Event 4 vs. Event 6) by three (Pair Type: pair type 1 vs. 2 vs. 3) within-measures ANOVA (n=32) revealed a significant main effect of Pair Type ($F(1.787,55.39) = 14.64$, $p < 0.001$, $\eta^2=3.176\%$). For Experiment 4 (d), a two (Pair Type: within vs. across) by two (Position Type: early vs. late) within-measures ANOVA (n=30) revealed a significant main effect of Pair Type ($F(1,29)=16.77$, $p< 0.001$,$\eta^2= 2.794\%$) and a main effect of Position Type ($F(1,29)=4.579$, $p=0.0409$, $\eta^2= 0.2654\%$). The boxes show the inter-quartile range (IQR) and the median. Whiskers in box plots represent the minimum and maximum in the dataset. Source data are provided as a Source Data file.

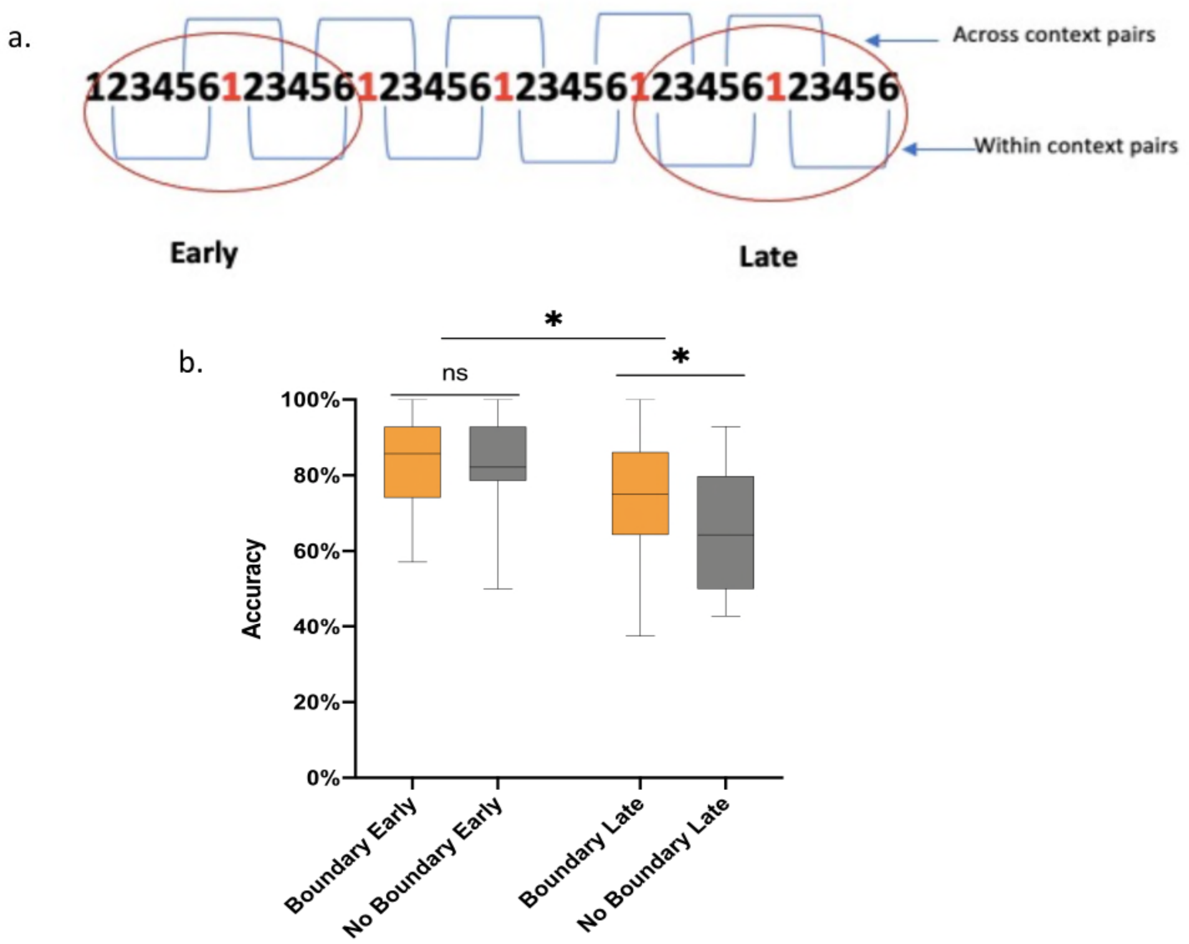

Supplementary Figure 3: **Primacy effect in the long sequence**. A. Schematic diagram showing the early and late positions in the long list for [matched] within-event items in Experiment 1. B. Box plots of the accuracy of temporal order memory for early and late positions for the boundary condition and no boundary condition (n=26). There was a significant Condition by Position interaction (F (1, 25) = 4.601, p=0.0419, η^2= 2.148%), a significant main effect of Condition (F (1, 25) = 5.291, p=0.0301, η^2=2.508%) and a significant main effect of Position (F (1, 25) = 15.11, p<0.001, η^2=14.26%). Simple effect analysis showed that in both conditions, there was a decrease in TOM accuracy from early to late positions (early vs. late position in the boundary condition: t(25)= 2.392, p= 0.0246, two-sided, q<0.05, FDR corrected for multiple comparisons, and in the no boundary condition: t(25)= 5.425, p <0.001, two-sided, q<0.05, FDR corrected for multiple comparisons). Critically, and consistent with our model predictions, TOM was significantly worse for late position in the no boundary condition compared to the boundary condition (boundary vs. no boundary for late position: t(25)=3.156, p=0.0041, two-sided, q<0.05, FDR corrected for multiple comparisons, while t(25) = 0.1222, p=0.9037, two-sided, q>0.05, FDR corrected for multiple comparisons for early position). The boxes show the inter-quartile range (IQR) and the median. Whiskers in box plots represent the minimum and maximum in the dataset. Source data are provided as a Source Data file.

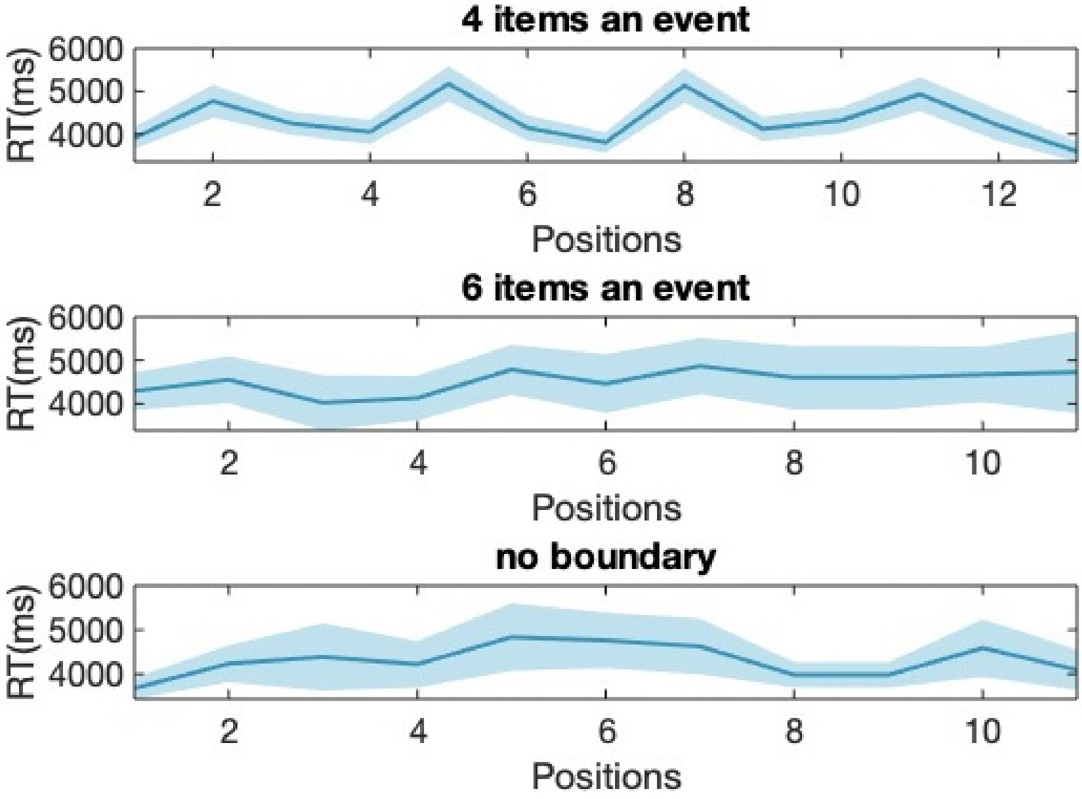

Supplementary Figure 4: Reaction time (RT) of recency judgments for pairs of items in Experiment 2 (4 items an event) and Experiment 1 (6 items an event and no boundary condition). Pairs were sorted according to the list position of the most recent item in each probed pair, such that pair 1 in the X-axis represents the probed pair in which the most distant item takes the earliest list position among those of all probed pairs. Shaded area represents the standard error of the mean. Source data are provided as a Source Data file.

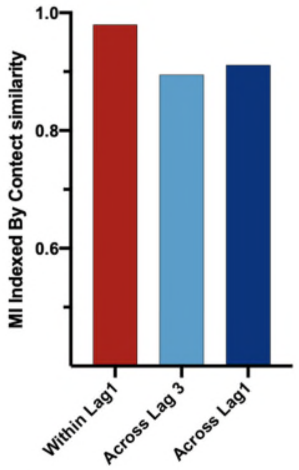

Supplementary Figure 5: The model outputs (i.e., average of 1000 iterations of model simulations) of Horner et al. (2016)[1]'s model regarding the boundary effect and the temporal distance effect. Horner et al. (2016)[1]'s model uses context similarity of the two probed items as the index for temporal order memory, with higher similarity corresponding to higher accuracy for temporal order memory (TOM). As shown here, this model captured correct boundary effect (i.e., better TOM for within-event pairs separated by one item than across-event pairs separated by one item), but incorrect temporal distance effect (i.e., worse TOM across-event pairs separated by three items than across-event pairs separated by one item, which is contrast with the empirical observations). The parameter values used in the simulation were drift rate = 0.01, shift rate = 0.08, as used in Horner et al. (2016)[1]. Source data are provided as a Source Data file.

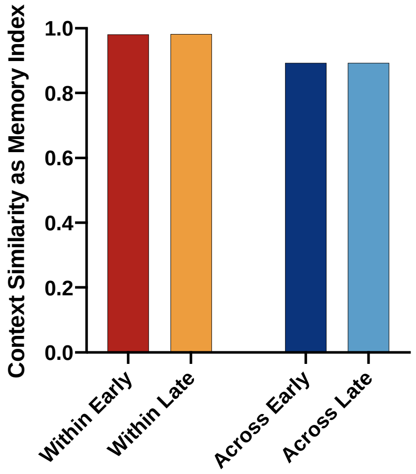

Supplementary Figure 6: The model outputs (i.e., average of 1000 iterations of model simulations) of Horner et al. (2016)[1]'s model regarding the local primacy effect. Horner et al. (2016)[1]'s model uses context similarity of the two probed items as the index for temporal order memory, with higher similarity corresponding to higher accuracy for temporal order memory (TOM). Model simulation shows no clear difference between pairs taking early and late event positions. Source data are provided as a Source Data file.

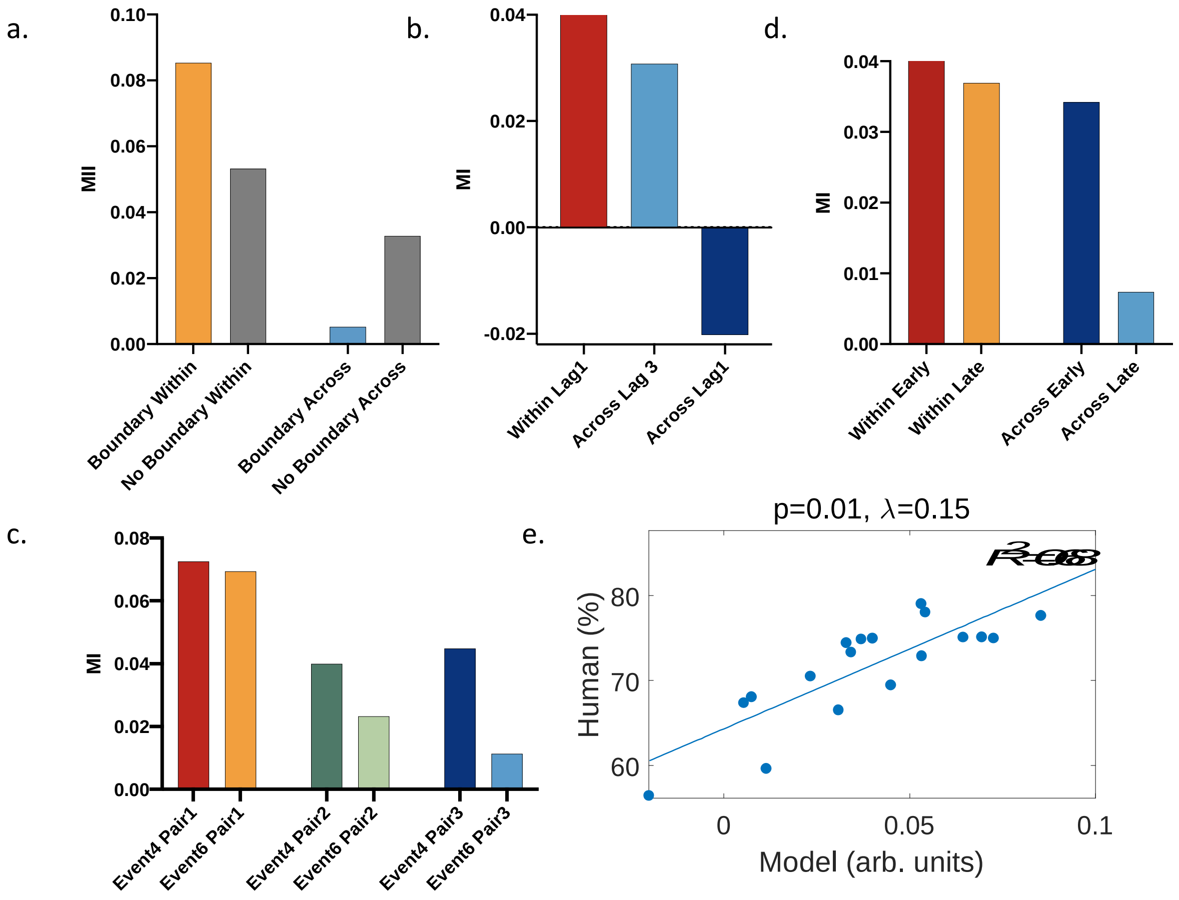

Supplementary Figure 7: Model results. a-d. Model outputs as quantified by our memory index (MI, i.e., d2-d1) for Experiments 1-4, when the drift rate (p) and reset rate (λ) were set as 0.1 and 0.15 respectively. e. Pearson correlation between model outputs shown in Fig. S7A-D and the corresponding group averaged behavioural results pooled together across experiments. The shaded area represents 95% confidence interval of the regression line. arb. units refers to arbitrary units. Source data are provided as a Source Data file.

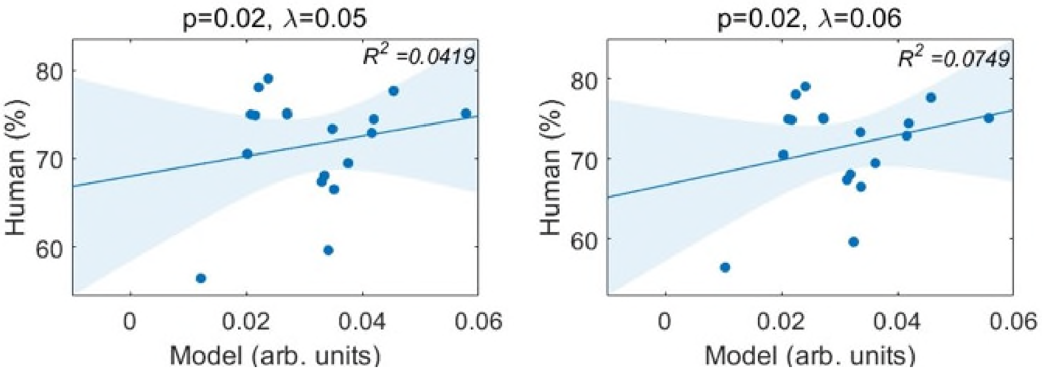

Supplementary Figure 8: Correlations between behavioural results and model outputs using two sets of parameter values. These figures show two examples when the model outputs could not account for the behavoural data (e.g., low R^2). The shaded area represents 95% confidence interval of the regression line. arb. units refers to arbitrary units. Source data are provided as a Source Data file.

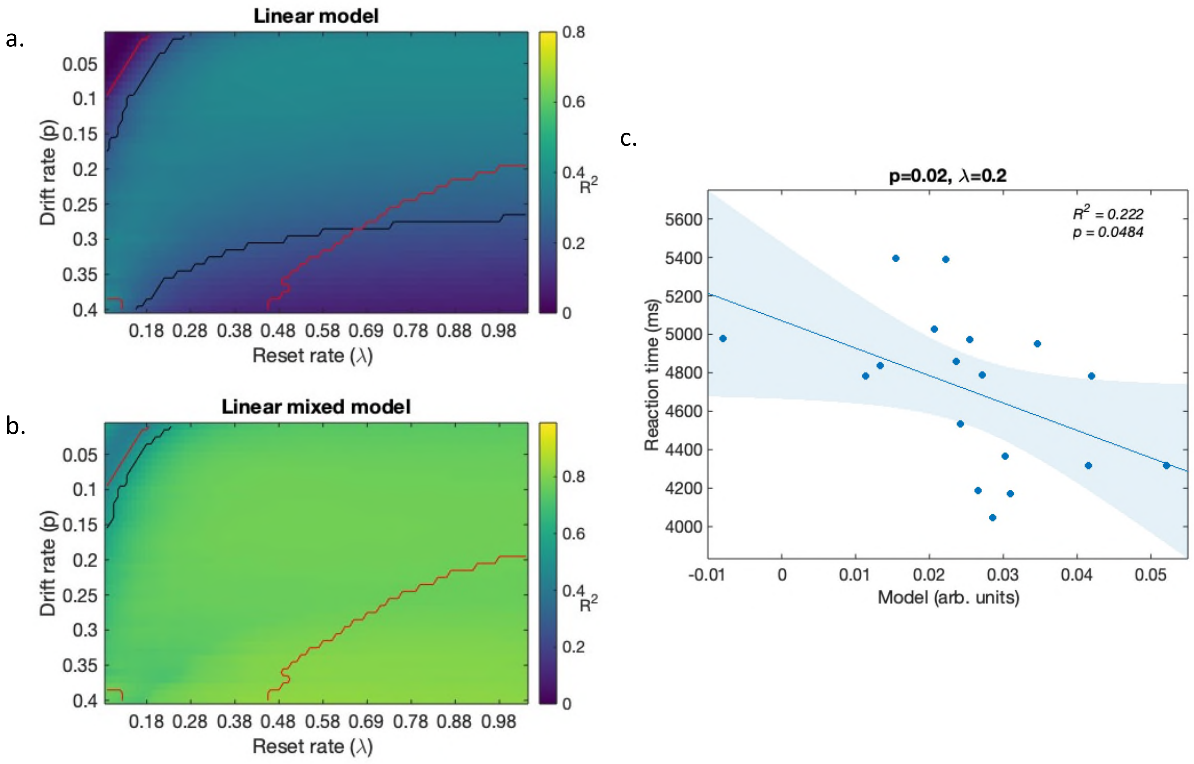

Supplementary Figure 9: Correlation between model outputs and reaction time (RT) under different parameter values. a. Explained variance (R^2) of RT by the model outputs, fitted with a generalized linear model. b. Explained variance (R^2) of RT by the model outputs, fitted with a generalized linear mixed model. c. Scatter plot of Pearson correlation between model outputs and RT under one parameter value. The shaded area in the scatter plot represents 95% confidence interval of the regression line. The black line in images indicates the significance threshold of p = 0.05, and the red line indicates the parameter values that yield model outputs which can recover all the effects in the four behavioural experiments. arb. units refers to arbitrary units. Source data are provided as a Source Data file.

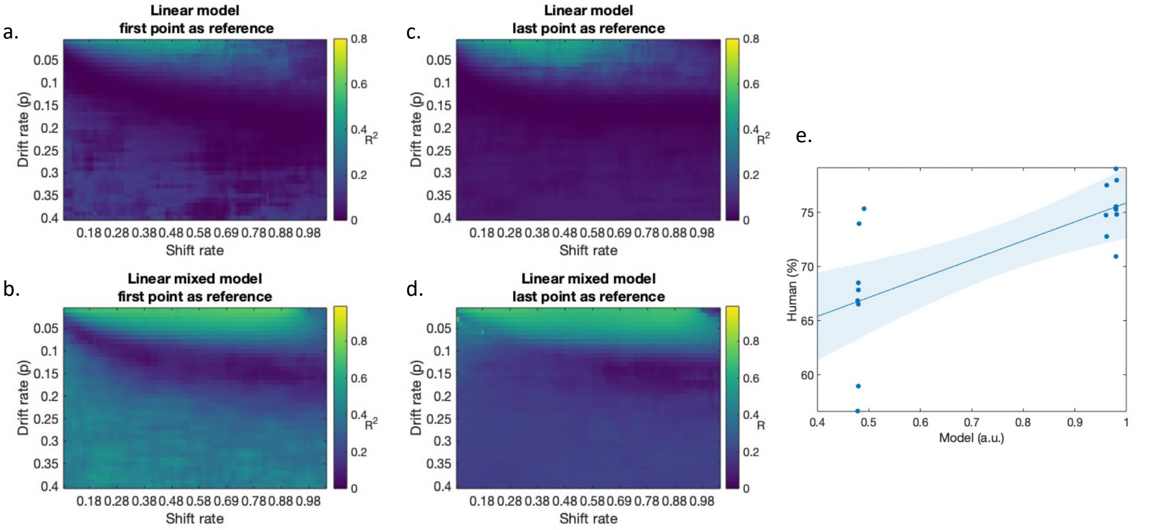

Supplementary Figure 10: Model outputs of Horner et al. (2016)[1]'s model in combination with our memory index. a-d. Explained variance (R^2) of behavioural results by model outputs, fitted with a generalized linear model or generalized linear mixed model. a-b used the first time point as the reference point for recency judgments; c-d used the last time point as the reference point for recency judgments. Overall, R^2 of Horner's model is smaller than that of the proposed model (see Fig. 5) across parameter values. e shows the correlation between the model outputs with the maximum R^2 in Supplementary Figure 3A and the behavioural data across experiments. As seen in e, the correlation was mainly driven by two clusters, within each of which the model outputs are not distinguishable for different experimental manipulations. The shaded area represents 95% confidence interval of the regression line. arb. units refers to arbitrary units. Source data are provided as a Source Data file.

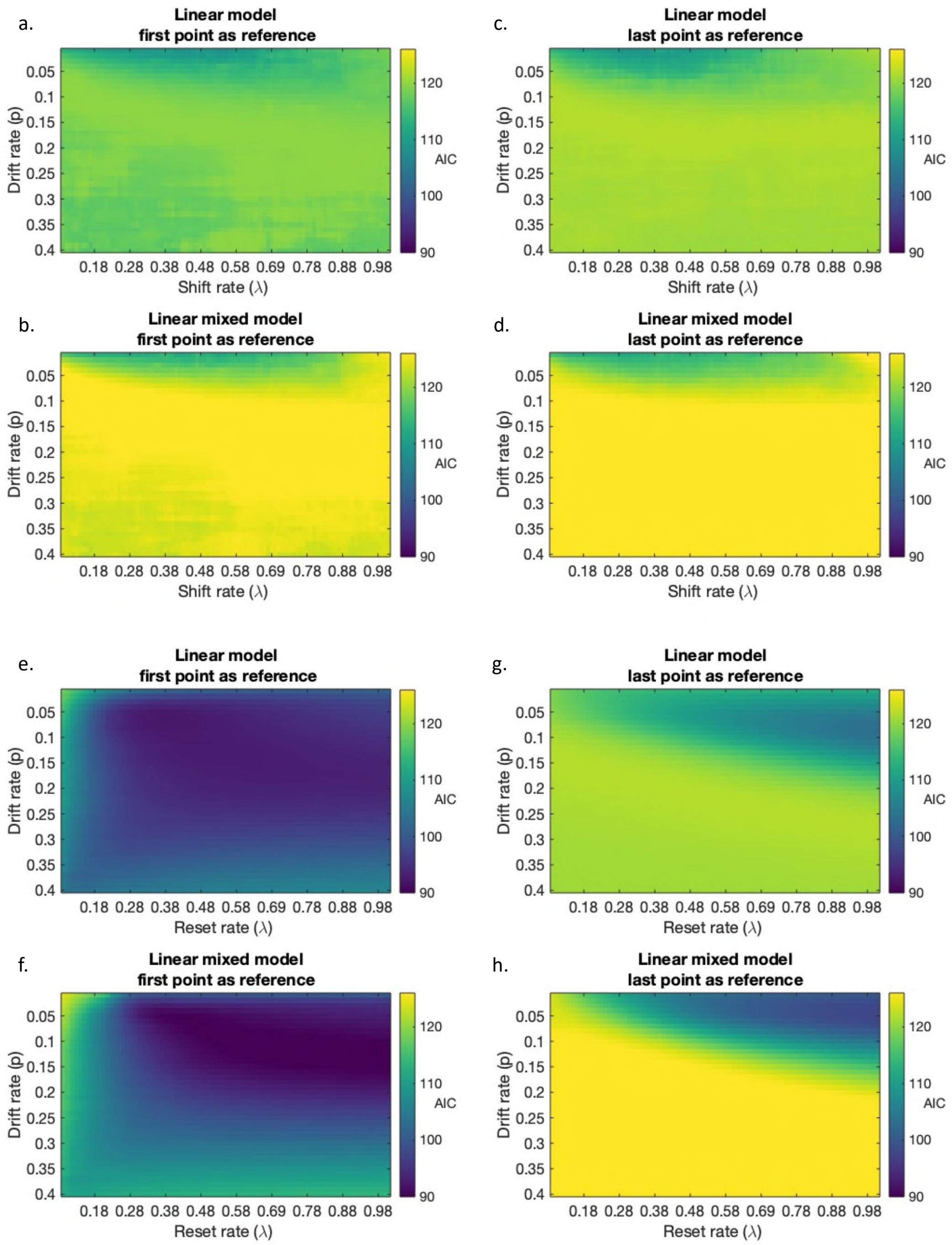

Supplementary Figure 11: The value of Akaike information criterion (AIC) of the fit of the model outputs to the empirical data for Horner et al. (2016)[1]'s model (a-d) and the proposed model (e-h), with a generalized linear model (a,b,e,f) and generalized linear mixed model (c, d, g, h). a-b & e-f used the first time point as the reference point for recency judgments; c-d & g-h used the last time point as the reference point for recency judgments. Overall, AIC is smallest for the proposed model using the first point as the reference among all models. Source data are provided as a Source Data file.

Supplementary References

1. Horner AJ, Bisby JA, Wang A, Bogus K, Burgess N. The role of spatial boundaries in shaping long-term event representations. *Cognition* **154**, 151-164 (2016).
