# Supplementary material for: Event boundaries shape temporal organization of memory by resetting temporal context
Source: Nat Commun. 2022 Feb 2;13:622. doi: 10.1038/s41467-022-28216-9 (PMC8810807; doi:10.1038/s41467-022-28216-9)
Supplement: Supplementary file 3 — Reporting summary [file 41467_2022_28216_MOESM3_ESM.pdf]

nature research

Corresponding author(s): Yi Pu
Last updated by author(s): Nov 24, 2021

Reporting Summary

Nature Research wishes to improve the reproducibility of the work that we publish. This form provides structure for consistency and transparency in reporting. For further information on Nature Research policies, see our Editorial Policies and the Editorial Policy Checklist.

Statistics

For all statistical analyses, confirm that the following items are present in the figure legend, table legend, main text, or Methods section.

n/a	Confirmed	
☐	☒	The exact sample size (n) for each experimental group/condition, given as a discrete number and unit of measurement
☐	☒	A statement on whether measurements were taken from distinct samples or whether the same sample was measured repeatedly
☐	☒	The statistical test(s) used AND whether they are one- or two-sided<br>*Only common tests should be described solely by name; describe more complex techniques in the Methods section.*
☐	☒	A description of all covariates tested
☐	☒	A description of any assumptions or corrections, such as tests of normality and adjustment for multiple comparisons
☐	☒	A full description of the statistical parameters including central tendency (e.g. means) or other basic estimates (e.g. regression coefficient) AND variation (e.g. standard deviation) or associated estimates of uncertainty (e.g. confidence intervals)
☐	☒	For null hypothesis testing, the test statistic (e.g. F, t, r) with confidence intervals, effect sizes, degrees of freedom and P value noted<br>*Give P values as exact values whenever suitable.*
☒	☐	For Bayesian analysis, information on the choice of priors and Markov chain Monte Carlo settings
☒	☐	For hierarchical and complex designs, identification of the appropriate level for tests and full reporting of outcomes
☐	☒	Estimates of effect sizes (e.g. Cohen's d, Pearson's r), indicating how they were calculated

Our web collection on statistics for biologists contains articles on many of the points above.

Software and code

Policy information about availability of computer code

Data collection	Visual stimuli were delivered and the participants' data were recorded using Presentation software (https://www.neurobs.com/).
Data analysis	Data analysis was performed using commercial software Prism (https://www.graphpad.com/scientific-software/prism/) and matlab.

For manuscripts utilizing custom algorithms or software that are central to the research but not yet described in published literature, software must be made available to editors and reviewers. We strongly encourage code deposition in a community repository (e.g. GitHub). See the Nature Research guidelines for submitting code & software for further information.

Data

Policy information about availability of data

All manuscripts must include a data availability statement. This statement should provide the following information, where applicable:

- Accession codes, unique identifiers, or web links for publicly available datasets
- A list of figures that have associated raw data
- A description of any restrictions on data availability

Source data is provided with the paper.

Field-specific reporting

Behavioural & social sciences study design

All studies must disclose on these points even when the disclosure is negative.

Study description	This study uses quantitative experiment methods.
Research sample	Young and healthy participants (age range: 18-38, males and females) were recruited from Frankfurt and its neighbouring areas. Frankfurt is a highly international city. Participants are from various ethnic and language backgrounds.
Sampling strategy	Participants were recruited and randomly assigned to experimental conditions. The minimum sample size of each experiment (N = 26) is based on a power analysis of a previous study (power=.8, alpha=.05).
Data collection	Data were collected using desktops in the lab. Researchers were not blind to study hypotheses. After given experimental instruction, participants independently conducted the experiment independently in a cabin.
Timing	Experiments were conducted between Feb. 2019 and Dec., 2019.
Data exclusions	6 participants were excluded from data analysis due to technical problems or unfinished tasks.
Non-participation	No participants declined to participate.
Randomization	Participants were randomly assigned to experimental conditions if applicable.

Reporting for specific materials, systems and methods

We require information from authors about some types of materials, experimental systems and methods used in many studies. Here, indicate whether each material, system or method listed is relevant to your study. If you are not sure if a list item applies to your research, read the appropriate section before selecting a response.

Materials & experimental systems

n/a	Involved in the study	
☒	☐	Antibodies
☒	☐	Eukaryotic cell lines
☒	☐	Palaeontology and archaeology
☒	☐	Animals and other organisms
☐	☒	Human research participants
☒	☐	Clinical data
☒	☐	Dual use research of concern

Methods

n/a	Involved in the study	
☒	☐	ChIP-seq
☒	☐	Flow cytometry
☒	☐	MRI-based neuroimaging

Human research participants

Policy information about studies involving human research participants

Population characteristics	See above.
Recruitment	Participants were recruited using our institute's electronic participant recruitment system and flyers posted on the University campus in Frankfurt. All participants gave their written informed consent and were compensated financially for their participation.
Ethics oversight	Ethics council of Max Planck Society

Note that full information on the approval of the study protocol must also be provided in the manuscript.
