# Supplementary material for: Event boundaries shape temporal organization of memory by resetting temporal context
Source: Nat Commun. 2022 Feb 2;13:622. doi: 10.1038/s41467-022-28216-9 (PMC8810807; doi:10.1038/s41467-022-28216-9)
Supplement: Supplementary file 4 — Source Data [file 41467_2022_28216_MOESM4_ESM.zip › SourceData/ReadMe.rtf]

The folder of ‘SourceData’ contains the following six parts, which contain all the data for figures 1-5 and supplementary figures S1-S11.  The excel file “sourceDataforEmpiricalData_Fig1to5.xlsx” contains all the empirical data for fig. 1-3 & 5e.The excel file “sourceDataSupplementaryData.xlsx” contains all the data for supplementary figures fig.S1-8The “sourceData_modelSimulation_boundaryVsnoBoundary.mat” file contains all the data for fig. 4b&c.The “sourceData_modelSimulation.mat” file contains the data for figure 5a-d, 6, fig.S11.The “sourceData_modelSimulation_RT.mat” contains the data for supplementary figure fig.S9.The “sourceData_forHorner'sModel.mat” contains data for supplementary figure fig.S10, fig.S11.***********************************************************************************************************************************************************************The ‘sourceData_modelSimulation.mat ‘contains simulation results (i.e., seq4, seq6, sq6_noBoundary, seq336_633_mean) for parameter values from p=0.01-0.4, with steps of 0.01, and λ0.05-1, with steps of 0.01. The first two dimensions of the data corresponding to each p and λrespectively. The last dimension corresponds to different experimental condition and the reference point used in model simulation (see below for details). Specifically, seq6 simulates the condition with 6 items per event, seq4simulates the condition with 4 items per event, seq 6_noBoundary simulates no boundary condition, seq336_633_mean simulates the last empirical experiment. 1-5 of the lasts dimension of seq6 represent simulations for W_lag3, A_lag3, W_early W_late, and A_late using the first context as the reference 1-6 of the last dimension of seq4 represent W_Lag1, A_Lag3, A_Lag1, W_early, W_late, A_early using the first context as the reference 1-2 of the last dimension of seq6_noBoundary represent seq6_no_W_lag3, seq6_no_A_lag3 using the first context as the reference 1-5 of the last dimension of seq336_633 represent W_early,  W_late, A_early,  sA_late, A_long  sing the first context as the reference 6-10 of the lasts dimension of seq6 represent simulations for W_lag3, A_lag3, W_early W_late, and A_late using the last context as the reference 7-12 of the last dimension of seq4 represent W_Lag1, A_Lag3, A_Lag1, W_early, W_late, A_early using the last context as the reference 3-4 of the last dimension of seq6_noBoundary represent seq6_no_W_lag3, seq6_no_A_lag3 using the last context as the reference 6-10 of the last dimension of seq336_633 represent W_early,  W_late, A_early,  sA_late, A_long  sing the last context as the reference gof_Rsq_fixed_first,  gof_Rsq_fixed_rdm_first, gof_Rsq_fixed_last, gof_Rsq_fixed_rdm_last contain R squared value of the model fit for fig 6a-d respectively.  AIC_fixed_first , AIC_rdm_first, AIC_fixed_last, AIC_rdm_last contains AIC value of the model fit for fig. S11e-h respectively**********************************************************************************************************************************************************************In “sourceData_modelSimulation_boundaryVsnoBoundary.mat” file, contexts of 36 time points were simulated. The similarity between each time point was calculated. The above process was iterated for 1000 times, constituting the 36 times 36 times 1000 similarity matrix. “corrs_boundary” contains the similarity matrix for boundary condition, and “corrs_noBoundary” contains the similarity matrix for no boundary condition.**********************************************************************************************************************************************************************In “sourceData_modelSimulation_RT.mat” file, got_Rsq_fixed_first and gof_Rsq_rdm_first contain the data for R squared value of the model fit for supplementary fig. S9a-b. The simulation data for Fig.S9c can be accessed in all_pairs_first(2,16,:), and the RT data can be accessed in all_bhv. ***********************************************************************************************************************************************************************The ‘sourceData_forHorner'sModel.mat ‘contains simulation results for Horner’s model in combination with the metric d2-d1 (i.e., Horner_seq4, Horner_seq6, Horner_ seq6_noBoundary, Horner_seq336_633_mean) for parameter values from p=0.01-0.4, with steps of 0.01, and sharp change from 0.05 to 1, with steps of 0.01. The first two dimensions of the data corresponding to each p and sharp change rate respectively. The last dimension corresponds to different experimental condition and the reference point used in model simulation (see below for details). 1-5 of the lasts dimension of Horner_seq6 represent simulations for W_lag3, A_lag3, W_early W_late, and A_late using the first context as the reference 1-6 of the last dimension of Horner_seq4 represent W_Lag1, A_Lag3, A_Lag1, W_early, W_late, A_early using the first context as the reference 1-2 of the last dimension of Horner_seq6_noBoundary represent seq6_no_W_lag3, seq6_no_A_lag3 using the first context as the reference 1-5 of the last dimension of Horner_seq336_633 represent W_early,  W_late, A_early,  sA_late, A_long  sing the first context as the reference 6-10 of the lasts dimension of Horner_seq6 represent simulations for W_lag3, A_lag3, W_early W_late, and A_late using the last context as the reference 7-12 of the last dimension of Horner_seq4 represent W_Lag1, A_Lag3, A_Lag1, W_early, W_late, A_early using the last context as the reference 3-4 of the last dimension of Horner_seq6_noBoundary represent seq6_no_W_lag3, seq6_no_A_lag3 using the last context as the reference 6-10 of the last dimension of Horner_seq336_633 represent W_early,  W_late, A_early,  sA_late, A_long  sing the last context as the reference gof_Rsq_fixed_first,  gof_Rsq_fixed_rdm_first, gof_Rsq_fixed_last, gof_Rsq_fixed_rdm_last contain R squared value of the model fit for fig.S10a-d respectively.  AIC_fixed_first , AIC_rdm_first, AIC_fixed_last, AIC_rdm_last contains AIC value of the model fit for fig. S11a-d respectively
